# Impact of COVID-19, cancer survivorship and patient-provider communication on mental health in the US Difference-In-Difference

**DOI:** 10.1038/s44184-023-00034-x

**Published:** 2023-08-30

**Authors:** Jiyeong Kim, Eleni Linos, Melanie S. Dove, Jeffrey S. Hoch, Theresa H. Keegan

**Affiliations:** 1 Department of Public Health Sciences, University of California Davis, Davis, CA USA; 2 Stanford Center for Digital Health, School of Medicine, Stanford, CA USA; 3 Program for Clinical Research & Technology, School of Medicine, Stanford University, Stanford, CA USA; 4 Division of Hematology and Oncology, University of California Davis Comprehensive Cancer Center, Sacramento, CA USA

**Keywords:** Epidemiology, Risk factors, Lifestyle modification, Preventive medicine, Depression, Anxiety

## Abstract

Poor mental health has been found to be more prevalent among those with cancer and is considered a public health crisis since COVID-19. This study assessed the impact of COVID-19 and cancer survivorship on mental health and investigated factors, including online patient-provider communications (OPPC; email/internet/tablet/smartphone), associated with poor mental health prior to and during the early COVID-19. Nationally representative Health Information National Trends Survey data during 2017–2020 (*n* = 15,871) was used. While the prevalence of poor mental health was high (40–42%), Difference-In-Difference analyses revealed that cancer survivorship and COVID-19 were not associated with poor mental health. However, individuals that used OPPC had 40% higher odds of poor mental health. Low socioeconomic status (low education/income), younger age (18–64 years), and female birth gender were also associated with poor mental health. Findings highlight the persistence of long-standing mental health inequities and identify that OPPC users might be those who need mental health support.

## Introduction

Poor mental health, including anxiety, depression, and psychological distress, affects individuals’ well-being and quality of life^1^. Poor mental health has been found to be more common among cancer survivors than those without a history of cancer due to disease-related concerns, including cancer recurrence, modified body image, or challenges in long-term healthcare needs^2^. It has been reported to negatively impact treatment adherence, self-management, and mortality among cancer survivors^3–6^. Approximately 25% to 40% of cancer survivors experienced poor mental health in 2019^7^. Previously, cancer survivors of Black/African American race/ethnicity, who were unmarried, with lower income, with lower education, who live in a rural residence, or who have low health literacy were reported to have poorer mental health^3,8–12^. As communications with healthcare providers play an important role in psychological distress management, optimal quality of patient-centered communication (PCC) style^13,14^ and online-based communications with providers^15–18^ have been shown to benefit managing poor mental health.

Under the unprecedented COVID-19 pandemic, psychosocial distress or depression increased in the general population^19–21^, as well as among cancer survivors^22–24^ worldwide. However, studies in the U.S. have observed mixed findings. Health Information National Trends Survey (HINTS) cross-sectional data reported that the prevalence of depression/anxiety slightly decreased among cancer survivors in the U.S. during COVID-19 (2019 vs. 2020), but did not compare estimates to adults without a history of cancer^25^. Alternatively, the COVID-19 Impact Study using 2020 cross-sectional data reported that cancer survivors had more mental health symptoms, including feeling nervous, anxious, hopeless, lonely, and depressed, than their non-cancer counterparts during COVID-19^26^. Last, a study using longitudinal data from 5 U.S. regions (Thinking and Living With Cancer) found that depression and anxiety worsened to a similar extent between breast cancer survivors ≥60 years of age and those without cancer during the pandemic^27^. Prior studies, to our knowledge, have not examined both the impact of COVID-19 and cancer survivorship on mental health, encompassing depression/anxiety and psychological distress. Additionally, chronic medical conditions have not been assessed for potential associations with poor mental health previously among cancer survivors during COVID-19^25^. Moreover, during the pandemic, PCC was significantly associated with cancer survivors’ mental health^28^, and online patient-provider communication (OPPC) became an essential channel to address psychological distress^29–31^. However, no studies have investigated the associations of PCC and OPPC with mental health accounting for sociodemographic and clinical factors.

Therefore, this study used HINTS data to assess changes in mental health before (2017–2019) and during COVID-19 (2020) in cancer survivors compared to adults without a history of cancer, taking into consideration patient-provider communication (PCC and OPPC). In addition, we examined the associations of the quality of PCC and OPPC with mental health by time period and cancer survivorship after accounting for sociodemographic and clinical factors. The findings of our study will identify those with poor mental health and inform targeted approaches to improve mental health outcomes.

## Methods

### Data source

Health Information National Trends Survey (HINTS), a nationally representative survey distributed and collected by the National Cancer Institute (NCI) was used for the study. HINTS is a self-administered, publicly available, cross-sectional survey. The present study used HINTS 5 Cycles 1, 2, 3, and 4 (2017–2020). Of note, the COVID-19 sample (2020) was collected from February to June 2020. The respondents of the survey questionnaires were non-institutionalized civilians 18 years and older in the United States. HINTS 5 Cycles 2, 3, and 4 have two geographic stratum: areas with low and high minority concentrations. HINTS 5 Cycle 1 had one more geographic stratum: an area in Central Appalachia. HINTS 5 was a single-mode mailed survey with a two-stage sampling design in Cycles 1, 2, and 4 and a double-mode design with a pilot push-to web survey in addition to the mailed survey in Cycle 3. HINTS 5 Cycle 3 was remediated and updated in March 2021 and we used the most recent version of HINTS data. This study followed the Strengthening the Reporting of Observational Studies in Epidemiology (STROBE) guidelines^32^. The total number of respondents in HINTS 5 Cycles 1–4 was 16,092. The average response rate was 33% (32.4% in Cycle 1 [*n* = 3285]; 32.4% in Cycle 2 [*n* = 3504]; 30.3% in Cycle 3 [*n* = 5438]; 36.7% in Cycle 4 [*n* = 3865])^33^. Among the total respondents, those who reported a history of cancer diagnosis were designated as cancer survivors (*n* = 2579) and the rest were considered as adults without a history of cancer (*n* = 13,292) after excluding those who missed reporting their history of cancer information (*n* = 221). We merged the four iterations (HINTS 5 Cycles 1–4) and obtained 200 replicate weights following the analytic suggestions from HINTS after confirming that there were no significant differences between variables of each iteration. The full-sample weights were applied to account for household-level base weight, non-response, and person-level initial weight^34^.

### Outcome

Mental health was measured by depression/anxiety diagnosis and psychological distress symptoms. To determine depression/anxiety diagnosis status, the question “Has a doctor or other healthcare professional ever told you that you had depression or anxiety disorder?” was used with the responses of “yes,” or “no.” To define psychological distress symptoms, the question “Over the past two weeks, how often have you been bothered by any of the following problems? (1) little interest in doing things, (2) feeling down, depressed, hopeless, (3) feeling nervous or anxious, (4) not being able to stop or control worrying” was used. These four questions were the same as those on the Patient Health Questionnaire (PHQ-4), a brief form commonly used to assess mental health^35,36^. The responses were measured by a Likert scale (1 = always, 2 = usually, 3 = sometimes, 4 = never). The scores from the four questions were summed to compute a total score, ranging from 4 (the worst) to 16 (the best). This total score was recorded as ‘yes’ for the score of 4–13 (mild/moderate/severe) and ‘no’ for the score of 14–16 (normal), following the PHQ-4’s cut-off approach^35^ to represent psychological distress. As a last step, we created a new mental health variable with depression/anxiety and total psychological distress score. If either depression/anxiety or the new psychological distress was ‘yes,’ then it was coded as poor mental health.

### Covariates

Patient-centered communications (PCC) was measured by the following seven questions that represent the main PCC functions that affect health outcomes, defined by the National Cancer Institute (NCI)^37^. “In your communication with all doctors, nurses, or other health professionals in the past 12 months, how often did they (1) give you the chance to ask health questions, (2) had the attention you needed to your feelings and emotions, (3) involve you in decisions about your health care as much as you wanted, (4) make sure you understood the things you needed to do to take care of your health, (5) explain things in a way you could understand, 6) spend enough time with you, (7) help you deal with uncertain feelings about your health or health care?”^37^ Responses for each question were measured on a Likert scale (1 = always, 2 = usually, 3 = sometimes, 4 = never). Responses to the seven questions were combined and recoded using the Likert scale numbers to generate a new continuous PCC outcome, ranging from a score of 0 (the least optimal, when all 7 questions were scored “never”) to a score of 100 (the most optimal, when all 7 questions were scored “always”)^38^. We also created a binary PCC variable with categories for optimal (when all 7 responses were ‘always’) and sub-optimal (any response of usually, sometimes, or never).

Online patient-provider communications (OPPC) were measured by 3 types of communication behaviors, as done previously^39^, using the following questions; (1) “In the past 12 months, have you used an email or the internet to communicate with a doctor or doctor’s office?”, (2) “Has your tablet or smartphone helped you in discussions with your healthcare provider?”, (3)“In the past 12 months, have you used your online medical record to securely message health care providers and staff?”. The response to each question was either “yes” or “no.” The tablet/smartphone and EHR questions were only asked to those who owned tablet computers/smartphones or used EHR at least once in the past 12 months. In this study, those who did not have a tablet/smartphone or use EHR once in the past 12 months were included in the no digital device use groups.

We chose sociodemographic factors as independent variables of this study based on the social determinants of health conceptual framework from the Healthy People 2030^40^: Age, birth gender, race/ethnicity, household income, educational attainment, marital status, employment status, health insurance type, a usual source of care, and rurality of residence. HINTS used Urban Rural Commuting Area (RUCA) to designate the rurality of residence of the survey respondents, which categorized census tracts using population density, urbanization, and commuting patterns developed by the United States Department of Agriculture^41^. Clinical factors included general health status, chronic medical conditions (diabetes, high blood pressure, heart disease, lung disease), time since cancer diagnosis, and diagnosed cancer type among survivors.

### Statistical analysis

We conducted descriptive analyses to present sociodemographic and clinical characteristics of cancer survivors and adults without a history of cancer prior to and during COVID-19 using means with standard errors (SE) or weighted percentages (%) with SE. The prevalence of poor mental health was estimated using a weighted percentage (%) with SE by sociodemographic and clinical characteristics. The mean PCC score with SE was estimated among cancer survivors and those without a history of cancer in pre-COVID-19 and COVID-19.

We conducted Differences-In-Differences (D-I-D) analysis in a weighted logistic regression model to identify the differences in the odds of poor mental health from pre- to during COVID, among cancer survivors compared to those without a history of cancer. The D-I-D of the odds of poor mental health was reported as an odds ratio (OR) with a 95% confidence interval (95% CI). D-I-D analyses were adjusted for age, gender, race/ethnicity, education, household income, general health status, and the chronic medical condition of lung disease because these variables were associated with mental health in prior studies^8,12,21,25^ or were confounders in our analyses (i.e., changed covariate estimates by more than 10%). The parallel trends assumption was tested quantitatively and also by visual inspection to assess if the trends of poor mental health were consistent in those with and without a history of cancer before the pandemic^42^. Additionally, we stratified the D-I-D analysis by PCC quality (optimal vs. sub-optimal) and OPPC (yes vs. no for Email/Internet communication, Tablet/Smartphone for discussion, EHR message) to identify changes in poor mental health by time period and patient-provider communication adjusting for the same covariates.

In addition, we developed a multivariable-adjusted weighted logistic regression model to examine the associations of history of cancer, COVID-19 time period, PCC, and OPPC with mental health after accounting for sociodemographic and clinical factors. Sensitivity analyses were conducted by separating the mental health outcome into psychological distress and chronic depression status to identify if there were differences in factors associated with each mental health outcome. We assessed the interactions of PCC (composite score) and three digital device use measures with both time periods and history of cancer. For these interaction assessments, we included interaction terms in multivariable logistic regression models. As above, variables included in the final models were associated with mental health in prior studies^8,12,21,25^ or were a potential confounder in our analyses. We performed imputation for any covariates with missingness, ranging from 0.5% to 22.8% (see footnotes of Table 1). Hot deck imputation was applied to account for missingness, which was also used for non-response by HINTS. As Cycle 3 did not contain employment status, it was not included in the model due to its large missingness (35% in employment status). Imputed data were used for all descriptive and regression analyses in SAS 9.4 (SAS studio 3.8, Cary, NC, USA). We did not perform adjustments for multiple testing as our study design was not confirmatory and planned to avoid the potential risk of increasing type II errors^43,44^. The statistical significance was determined at *p* < 0.05.

**Table 1:** Sociodemographic and clinical characteristics of cancer survivors and adults without a history of cancer by pre-COVID-19 and early COVID-19 time period (HINTS 5 2017–2020).

	Cancer survivors		Adults without a history of cancer	
	Pre-COVID-19 *N* = 1953^a^	COVID-19 *N* = 626^a^	Pre-COVID-19 *N* = 10,124^a^	COVID-19 *N* = 3168^a^
	Weighted % (SE)	Weighted % (SE)	Weighted % (SE)	Weighted % (SE)
Sociodemographic characteristics				
Age (years)				
18–34	4.8 (1.5)	3.1 (1.2)	24.6 (0.8)	28.3 (1.1)
35–49	10.8 (1.2)	16.2 (2.9)	27.9 (0.8)	26.4 (1.2)
50–64	33.2 (1.7)	32.1 (3.1)	30.2 (0.6)	27.4 (1.0)
65–74	25.6 (1.3)	24.6 (2.3)	10.4 (0.2)	10.7 (0.3)
≥ 75	25.6 (1.2)	24.0 (2.0)	7.0 (0.2)	7.3 (0.2)
Gender				
Female	57.2 (1.7)	56.9 (3.2)	50.6 (0.2)	50.8 (0.4)
Male	42.8 (1.7)	43.1 (3.2)	49.4 (0.2)	49.2 (0.4)
Race/ethnicity				
Non-Hispanic White	79.0 (1.6)	82.1 (2.1)	64.8 (0.3)	62.6 (0.5)
Non-Hispanic Black/African American	8.0 (1.3)	8.4 (1.7)	10.7 (0.2)	11.0 (0.4)
Hispanic	9.0 (1.2)	6.5 (1.6)	16.2 (0.2)	17.3 (0.2)
Non-Hispanic Asian	1.7 (0.4)	1.4 (0.5)	5.3 (0.2)	5.5 (0.3)
Others	2.4 (0.5)	1.7 (0.8)	3.0 (0.2)	3.6 (0.3)
Education				
Less Than High School	7.1 (1.2)	6.0 (1.4)	8.3 (0.5)	8.1 (0.9)
High School Graduate	26.6 (1.6)	28.9 (2.5)	22.4 (0.5)	21.7 (1.0)
Some College	38.5 (1.7)	36.0 (2.5)	37.7 (0.5)	39.1 (1.0)
College Graduate or More	27.8 (1.3)	29.1 (2.7)	31.7 (0.2)	31.1 (0.5)
Household income				
<$20,000	15.4 (1.4)	19.3 (2.6)	17.3 (0.7)	14.4 (0.9)
$20,000 to <$35,000	15.5 (1.3)	11.3 (2.0)	11.5 (0.6)	11.3 (0.8)
$35,000 to <$50,000	15.1 (1.8)	15.0 (2.1)	13.4 (0.6)	12.1 (0.9)
$50,000 to <$75,000	19.6 (1.5)	19.2 (2.7)	18.5 (0.7)	18.3 (1.5)
≥$75,000	34.5 (1.7)	35.2 (2.6)	39.2 (0.8)	44.0 (1.7)
Marital status^b^				
Married	61.2 (1.7)	63.6 (2.8)	54.0 (0.4)	54.5 (0.6)
Not married	38.8 (1.7)	36.4 (2.8)	46.0 (0.4)	45.3 (1.3)
Rurality				
Metropolitan	82.9 (1.3)	78.8 (2.1)	84.8 (0.6)	88.1 (0.8)
Micropolitan	9.9 (1.0)	11.3 (2.0)	9.1 (0.5)	7.4 (0.9)
Small town	3.6 (0.6)	4.9 (1.6)	3.3 (0.3)	3.1 (0.5)
Rural	3.6 (0.6)	5.0 (1.1)	2.9 (0.3)	1.4 (0.3)
Health insurance type				
Employment and private	33.0 (1.8)	35.2 (3.0)	54.3 (0.8)	54.5 (1.4)
Medicare	32.9 (1.4)	36.6 (2.6)	14.8 (0.5)	14.9 (0.8)
Medicaid	14.3 (1.7)	14.8 (2.3)	16.4 (0.7)	15.2 (0.9)
Tricare, VA, IHS	10.3 (1.0)	5.7 (1.1)	6.0 (0.4)	6.6 (0.5)
Others	9.6 (0.9)	7.8 (1.7)	8.5 (0.5)	8.9 (0.8)
Usual source of care				
Yes	83.8 (1.2)	85.7 (2.2)	63.8 (0.8)	60.6 (1.3)
No	16.2 (1.2)	14.3 (2.2)	36.2 (0.8)	39.4 (1.3)
Clinical characteristics				
General health status				
Excellent/good	74.1 (1.6)	76.3 (2.5)	85.3 (0.6)	86.8 (0.9)
Fair/poor	25.9 (1.6)	23.7 (2.5)	14.7 (0.6)	13.2 (0.9)
Chronic medical condition				
Diabetes	23.9 (1.6)	25.3 (2.5)	16.5 (0.6)	17.4 (1.1)
High blood pressure	54.4 (1.7)	54.7 (3.1)	35.0 (0.7)	34.5 (1.0
Heart disease	15.8 (1.4)	12.9 (1.7)	6.9 (0.3)	7.8 (0.7)
Lung disease	16.3 (1.1)	18.8 (2.3)	11.1 (0.4)	12.1 (0.7)
Time since diagnosis				
<1 year	13.3 (1.3)	15.6 (2.6)	–	–
2–5 years	21.5 (1.3)	17.5 (2.3)	–	–
6–10 years	18.4 (1.4)	19.8 (2.2)	–	–
≥11 years	46.8 (1.9)	48.1 (3.0)	–	–
Cancer type^c^				
Breast	12.7 (1.1)	14.9 (2.6)	–	–
Cervical	6.6 (1.0)	7.2 (1.9)	–	–
Prostate	6.3 (0.7)	6.7 (1.2)	–	–
Colon	3.9 (0.7)	3.7 (0.7)	–	–
Lung	2.1 (0.5)	1.0 (0.4)	–	–
Skin	25.5 (1.6)	22.6 (2.2)	–	–
Melanoma	3.8 (0.6)	8.3 (2.0)	–	–
Multiple cancers	17.5 (1.2)	13.4 (1.8)	–	–
Other cancers	21.6 (1.9)	22.2 (2.7)	–	–
Online PPC				
Email/Internet communication	60.6 (1.8)	48.7 (3.1)	61.7 (0.8)	53.1 (1.3)
Tablet/smartphone for discussion	69.9 (1.8)	60.8 (3.2)	69.0 (0.8)	64.9 (1.5)
EHR message	80.5 (1.3)	69.9 (3.1)	79.2 (0.6)	77.8 (1.2)
PCC composite score^d^				
Mean (SE)	70.2 (1.1)	71.0 (2.0)	60.6 (0.7)	64.3 (1.2)

*VA* Veterans Affairs, *IHS* Indian Health Services.

^a^Missingness of covariates ranged from 0.48% to 22.84%. Covariates with any missing values were imputed in Table 1.

^b^Marital status (married or living with a romantic partner as a married vs. not married including divorced, widowed, separated, single/never been married).

^c^Less prevalent cancer types were recoded as others (bladder, bone, endometrial, head and neck, leukemia/blood, liver, lymphoma, oral, ovarian, pancreatic, pharyngeal, rectal, renal, stomach cancer, and unknown cancer).

^d^PCC score ranges from 0 (sub-optimal) to 100 (optimal), higher is better.

### Ethics statement

The current study used the publicly available national survey data, Health Information National Trends Survey (HINTS). HINTS is a deidentified dataset, and this study is a secondary analysis of the deidentified survey. Because the human subject was not involved in this study, written consent is not applicable. Given that identifiable information was not included, this study was deemed exempt from review by the Institutional Review Board at the University of California, Davis.

### Reporting summary

Further information on research design is available in the Nature Research Reporting Summary linked to this article.

## Results

### Sociodemographic and clinical characteristics of the study population

Table 1 shows population characteristics of cancer survivors and adults without a history of cancer, before and during COVID-19. Cancer survivors were older, with 51% of cancer survivors aged 65 or older compared to 17% of adults without a history of cancer. Among cancer survivors, 36% were employed (vs. 58% in those without a history of cancer), 33% had private or employment-based insurance (vs. 54%), 84% had a usual source of care (vs. 63%), 25% reported poor general health status (vs. 14%), and 15–54% had chronic health conditions (vs. 7–35%). All three types of OPPC were similar between groups, yet differed by time, use was lower during COVID-19. PCC score was higher among cancer survivors (mean 70 vs. 62). There were no differences by time period by gender, race/ethnicity, or education.

### Prevalence of poor mental health

**Fig. 1:**
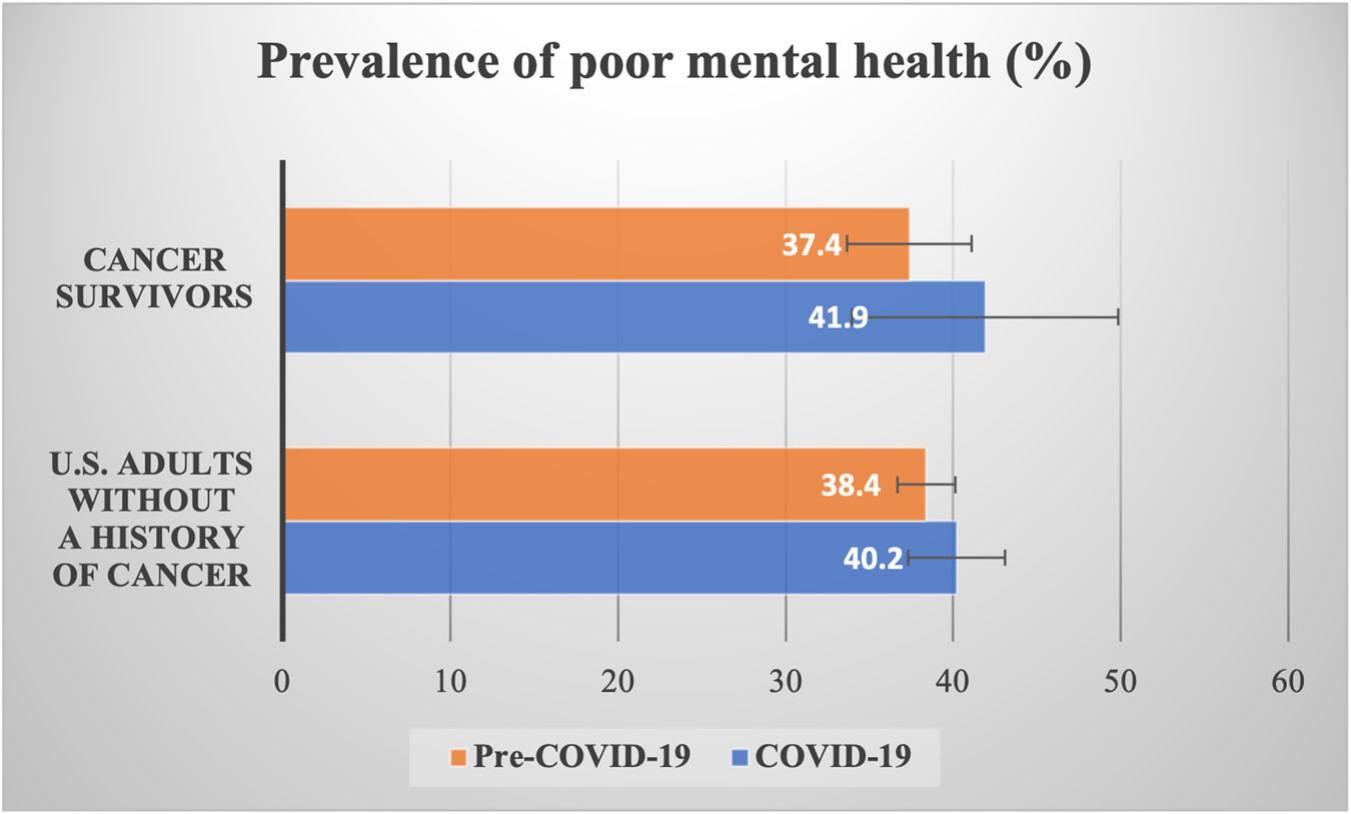
Prevalence of poor mental health in pre-COVID-19 (2017–2019) and COVID-19 (2020). Poor mental health was determined by either having depression/anxiety or psychological distress. Described in weighted percent (%) with 95% CI (error bars) among cancer survivors (*N* = 2449) and U.S. adults without a history of cancer (*N* = 12,791).

The prevalence of poor mental health increased from pre-COVID-19 to during COVID-19 in both cancer survivors (by 4.5%) and adults without a history of cancer (by 1.8%) (Fig. 1). During COVID-19, the prevalence of poor mental health during COVID-19 was similar for cancer survivors (41.9%) and adults without a history of cancer (40.2%). We observed the prevalence of poor mental health differed by sociodemographic and health status factors. Younger adults (35–49 years), females, least educated (less than high school), unmarried, those with low income (<$50,000), had Medicaid, had fair/poor health condition, had a chronic disease, or used a tablet/smartphone to communicate with providers had higher than the average prevalence of poor mental health in both groups and time periods (Supplement Table 1).

### Impact of early COVID-19 on mental health

The D-I-D analysis revealed that the changes in poor mental health prior to and during COVID-19 among cancer survivors compared to adults without a history of cancer were not significantly different (Table 2), overall or stratified by PCC or online PPC. When we stratified by PPC (optimal vs. sub-optimal), we observed that the odds of poor mental health significantly increased from pre-COVID-19 to during COVID-19 in adults without a history of cancer among those who had optimal PCC (OR = 1.32, 95% CI 1.00–1.15). Among cancer survivors, there was also an increase among adults with optimal PCC, but it was not statistically significant (OR = 1.31, 95% CI 0.76–2.25).

**Table 2:** Changes in poor mental health prior to (2017–2019) and during COVID-19 (2020) among cancer survivors by PCC quality and Online PPC.

			Cancer survivors			U.S. Adults without a history of cancer		
	Pre-COVID-19 (odds)	COVID-19 (odds)	Difference (aOR, 95% CI)	Pre-COVID-19 (odds)	COVID-19 (odds)	Difference (aOR, 95% CI)	Difference-In-Difference^a^ (aOR, 95% CI)	*p*-value
Overall^b^	0.93	1.13	1.21 (0.83–1.76)	0.87	0.98	1.14 (0.97–1.33)	1.07 (0.71–1.60)	0.76
PCC^c^								
Optimal	0.79	1.03	1.31 (0.76–2.25)	0.68	0.90	1.32 (1.00–1.15)^*^	0.99 (0.53–1.81)	0.96
Sup-optimal	0.99	1.13	1.14 (0.73–1.79)	0.99	1.03	1.04 (0.87–1.24)	1.10 (0.68–1.77)	0.71
Online PPC								
Email/Internet communication	1.03	1.16	1.13 (0.67–1.92)	1.06	1.20	1.13 (0.91–1.41)	1.00 (0.57–1.75)	0.99
No Email/Internet communication	0.85	1.06	1.26 (0.82–1.93)	0.79	0.86	1.09 (0.89–1.34)	1.15 (0.71–1.86)	0.56
Tablet/Smartphone for discussion	1.21	1.40	1.15 (0.61–2.18)	1.12	1.23	1.10 (0.85–1.41)	1.05 (0.52–2.11)	0.88
No Tablet/Smartphone for discussion	0.84	0.99	1.17 (0.78–1.77)	0.83	0.95	1.14 (0.94–1.38)	1.03 (0.66–1.62)	0.90
EHR message	0.76	0.65	0.85 (0.46–1.57)	0.70	0.87	1.24 (0.94–1.65)	0.68 (0.35–1.32)	0.25
No EHR message	0.97	1.33	1.38 (0.94–2.03)	0.92	1.00	1.09 (0.91–1.31)	1.26 (0.83–1.93)	0.28

*aOR* adjusted odds ratio, *CI* confidence interval.

^a^Refers to changes in the odds of poor mental health among cancer survivors compared to those with or without a history of cancer during the early COVID-19 pandemic in the generalized linear model using inverse link function to estimate the differences in differences log odds, adjusting for a history of cancer, time period, age, birth gender, education, race/ethnicity, household income, general health status, and chronic disease (lung disease).

^b^Total *n* = 15,240, the parallel trends assumption was met in pre-COVID-19 checked by visual inspection and quantitative test.

^c^Optimal PCC = When all 7 PCC questions were answered ‘always’.

^*^Statistically significant (*p* < 0.05).

### Factors associated with poor mental health

We did not observe interactions of PCC or digital device use measures with either history of cancer or a time period. Therefore, Table 3 shows factors associated with poor mental health in a multivariable model with cancer survivors and adults without cancer during both time periods. In multivariable models, individuals who used email/internet (OR = 1.39, 1.20–1.60) or tablet/smartphones (OR = 1.39, 1.21–1.59) to communicate with providers were more likely to have poor mental health. The odds of poor mental health were not associated with EHR message use or PCC composite score. In addition, a history of cancer (OR = 1.04, 0.88–1.23 vs. no) and the early COVID-19 pandemic (OR = 1.09, 0.94–1.27 vs. pre-COVID-19 2017–2019) were not associated with poor mental health. However, other health conditions were associated with poor mental health. Those with excellent/good general health status (vs. fair poor) were less likely to have poor mental health. Adults with chronic lung disease (OR = 1.72, 1.47–2.02) were 1.7 times as likely to have poor mental health than those without chronic lung disease.

**Table 3:** Factors associated with poor mental health in cancer survivors and adults without a history of cancer (HINTS 2017–2020).

	Poor mental health^a^		Psychological distress		Chronic depression	
	aOR^b^ (95% CI) *N* = 15,240	*p*-value	aOR^b^ (95% CI) *N* = 15,412	*p*-value	aOR^b^ (95% CI) *N* = 15,870	*p*-value
History of cancer		0.66		0.49		0.13
Yes	1.04 (0.88–1.23)		1.07 (0.89–1.27)		0.87 (0.72–1.04)	
No	Reference		Reference		Reference	
Time period		0.23		0.37		0.74
COVID-19 (2020)	1.09 (0.94–1.27)		1.07 (0.92–1.26)		1.03 (0.88–1.20)	
Pre-COVID-19 (2019–17)	Reference		Reference		Reference	
PCC^d^						
Per 10-unit composite score	1.00 (0.99–1.02)	0.75	0.98* (0.96–0.995)	0.01	1.05* (1.03–1.07)	<0.0001
Online PPC						
Email	1.39* (1.20–1.60)	<0.0001	1.31* (1.12–1.53)	0.0008	1.38* (1.17–1.61)	<0.0001
No Email	Reference		Reference		Reference	
Tablet	1.39* (1.21–1.59)	<0.0001	1.39* (1.20–1.61)	<0.0001	1.36* (1.17–1.59)	0.0001
No Tablet	Reference		Reference		Reference	
EHR	1.01 (0.87–1.17)	0.88	0.92 (0.78–1.08)	0.30	1.22* (1.03–1.46)	0.02
No EHR	Reference		Reference		Reference	
Age (years)						
18–34	3.25* (2.56–4.11)	<0.0001	3.00* (2.34–3.85)	<0.0001	3.06* (2.27–4.13)	<0.0001
35–49	2.58* (2.08–3.21)	<0.0001	1.90* (1.51–2.40)	<0.0001	3.13* (2.40–4.07)	<0.0001
50–64	1.98* (1.64–2.39)	<0.0001	1.57* (1.29–1.92)	<0.0001	2.33* (1.84–2.96)	<0.0001
65–74	1.16 (0.96–1.40)	0.14	0.90 (0.73–1.10)	0.30	1.49* (1.18–1.89)	0.001
≥75	Reference		Reference		Reference	
Gender						
Female	1.59* (1.40–1.80)	<0.0001	1.41* (1.22–1.62)	<0.0001	1.79* (1.55–2.08)	<0.0001
Male	Reference		Reference		Reference	
Race/Ethnicity						
NH White	Reference		Reference		Reference	
NH Black/African American	0.62* (0.51–0.75)	<0.0001	0.72* (0.58–0.90)	0.004	0.53* (0.42–0.67)	<0.0001
Hispanic	0.68* (0.56–0.82)	<0.0001	0.78* (0.63–0.95)	0.02	0.55* (0.45–0.68)	<0.0001
NH Asian	0.54* (0.39–0.76)	0.0003	0.67* (0.47–0.95)	0.02	0.31* (0.20–0.48)	<0.0001
Others	0.88 (0.60–1.28)	0.49	0.87 (0.60–1.25)	0.44	0.92 (0.63–1.35)	0.67
Education						
<High School	Reference		Reference		Reference	
High School Graduate	0.78 (0.59–1.02)	0.06	0.76 (0.57–1.01)	0.06	0.73 (0.52–1.01)	0.06
Some College	0.82 (0.63–1.06)	0.13	0.80 (0.61–1.05)	0.11	0.86 (0.63–1.17)	0.33
College Grad or More	0.72* (0.56–0.94)	0.02	0.64* (0.48–0.85)*	0.002	0.78 (0.55–1.09)	0.15
Household income						
<$20,000	Reference		Reference		Reference	
$20,000 to <$35,000	0.63* (0.49–0.79)	0.0001	0.62* (0.49–0.79)	0.0001	0.66* (0.52–0.83)	0.0005
$35,000 to <$50,000	0.57* (0.45–0.72)	<0.0001	0.58* (0.45–0.74)	<0.0001	0.60* (0.47–0.77)	<0.0001
$50,000 to <$75,000	0.42* (0.34–0.52)	<0.0001	0.46* (0.36–0.58)	<0.0001	0.44* (0.35–0.56)	<0.0001
≥$75,000	0.37* (0.30–0.45)	<0.0001	0.40* (0.32–0.50)	<0.0001	0.39* (0.31–0.49)	<0.0001
General health status						
Excellent/good	0.31* (0.26–0.37)	<0.0001	0.28* (0.24–0.33)	<0.0001	0.35* (0.29–0.43)	<0.0001
Fair/poor	Reference		Reference		Reference	
Chronic medical condition						
Lung disease	1.72* (1.47–2.02)	<0.0001	1.43* (1.21–1.71)	<0.0001	1.83* (1.55–2.17)	<0.0001
No lung disease	Reference		Reference		Reference	

^a^Poor mental health includes both psychological distress and chronic depression.

^b^Adjusted for all variables in the table.

^c^PCC score ranges from 0 (sub-optimal) to 100 (optimal), higher is better.

^*^p < 0.05.

Demographic factors associated with poor mental health included younger populations (ORs = 1.98–3.25; 18–64 vs. ≥75 years) and females (OR = 1.59, 1.39–1.80). Non-Hispanic Black/African Americans (OR = 0.62, 0.51–0.75), Hispanics (OR = 0.68, 0.56–0.82), and non-Hispanic Asians (OR = 0.54, 0.39–0.75) were less likely to have poor mental health than non-Hispanic White populations. More educated individuals (OR = 0.72, 0.56–0.94 college graduate or more vs. less than high school) were less likely to have poor mental health. Individuals with the lowest income (<$20,000) were approximately 2-3 times as likely to have poor mental health than those with higher income. Sensitivity analyses revealed that the factors associated with psychological distress and chronic depression were similar to poor mental health, except for PCC and EHR OPPC (Table 3). The odds of psychological distress decreased by 2% per 10-unit PCC composite score increase, while the odds of chronic depression status increased by 5% per 10-unit PCC score increase. Using EHR to communicate with providers was associated with chronic depression (OR = 1.22, 1.03–1.46), but no association was shown with poor mental health.

## Discussion

We examined the impact of COVID-19 and cancer survivorship on poor mental health and factors associated with poor mental health prior to and during the early COVID-19 pandemic using a nationally representative survey. The prevalence of poor mental health increased to a similar extent in both those with and without a history of cancer from pre-pandemic to early COVID-19, where the prevalence was high at approximately 40%. However, neither the COVID-19 pandemic nor cancer survivorship was associated with poor mental health during the early COVID-19 in the U.S. Notably, we found that OPPC use (email/internet and tablet/smartphone) was significantly associated with poor mental health, suggesting that active digital device users might be those who need mental health supports. In addition, our study identified subgroups of adults, defined by sociodemographic (younger age, females, lower income/education) and clinical (chronic lung disease or poor general health) factors, who were more likely to experience poor mental health. Our findings shed light on populations more likely to experience poor mental health and opportunities for targeted interventions to prevent further mental health inequities in the U.S.

Our findings showed that the prevalence of poor mental health increased during COVID-19 to a similar degree among cancer survivors and those without a history of cancer. Our findings of an increase in poor mental health aligned with the longitudinal study (2014–2020) among breast cancer survivors 60 years and older and adults without a history of cancer from 5 U.S. regions^27^. Despite the previous concerns that COVID-19-related situations (e.g., delayed cancer care, fear of disease progression) would disproportionately impact the mental health status of cancer survivors during COVID-19^45,46^, the prevalence did not differ between cancer survivors and those without a history of cancer. This highlights that there were concerns that might have led to poorer mental health in those without a history of cancer as well. Our findings of a high prevalence of poor mental health among U.S. adults during the early pandemic align with the findings of a national survey in April 2020 that observed 52% had mild or severe depression^21^. Thus, our findings contribute to the evidence of the unusually high prevalence of poor mental health during the early pandemic that will need to be carefully monitored post-pandemic.

In our study, communication through email/internet and tablet/smartphone with health providers was associated with poor mental health after accounting for PCC quality and sociodemographic and clinical factors. The three types of digital devices we considered involve different levels of digital fluency and experience. Specifically, email/internet communications could refer to a lower and general level of digital fluency, whereas discussions with tablet/smartphone require a higher level of digital fluency. On the other hand, messaging via EHR, which was only associated with chronic depression, demands being digitally engaged with the healthcare system. In previous literature, those with poor mental health were more likely to seek online activities for health (e.g., participate in online health discussion forums, and watch health-related videos)^47^. Given OPPC could be a part of online activity, it is possible that those with existing poor mental health were engaged more in OPPC. However, this interpretation needs caution as it is also possible that heavy online activities, which could have increased OPPC as well, led to poor mental health^48^. Although we cannot confirm the directions of associations observed in this cross-sectional study, our findings signaled that digital device-based communications could be a tool and provide additional opportunities to care for individuals with mental distress. Suggested interventions could include related stakeholders (e.g., healthcare clinics and public health practitioners) widely informing the public about the available digital device-based communication channels for those with mental distress^49^. Potentially, social media could also play a role in the dissemination of relevant information and online communication options^47^.

We observed that sub-optimal PCC was associated with poor psychological distress, which aligned with previous findings among cancer survivors during COVID-19^28^. This may highlight the role of PCC as a potential channel to address psychological distress^28^. However, notably, more optimal PCC was associated with chronic depression in our study. While further studies are needed to understand the factors underlying this association, it might have related to more encounters with providers (e.g., for depression treatment or comorbidity care) among those with chronic depression, as frequent office visits have shown association with optimal PCC, previously^37^.

Consistent with prior studies^21,25,26,50,51^, we also found that lower education level and income were associated with poor mental health. Overall wealth also has been associated with resilience during COVID-19, with those with resilience having lower odds of depression and anxiety in a longitudinal study^52^. In addition, COVID-19 pandemic-related loss of employment income was associated with worsened mental health^53^. Multifaceted approaches will need to be considered to relieve the poor mental health of those with low SES and address the root cause of the issue in the long-term^50^. Approaches could include partnering with already available community programs (e.g., Special Supplemental Nutrition Program for Women, Infants, and Children, WIC) or local governments (e.g., State Employment Development Department, EDD) to reach out to those in need, including low-income families or those who experienced unemployment during the pandemic, to gauge the mental health care needs and design tailored mental health care interventions^54,55^.

We also found that younger individuals and females were more likely to have poor mental health, which aligned with the previous literature^12,25,26^. These consistent associations highlight the need for targeted interventions for younger individuals and females. A prior study found that younger individuals had a lower level of resilience and poor sleep quality, mediators for depression and anxiety symptoms, and were more vulnerable to perceived stress, which was strongly associated with depression and anxiety symptoms during COVID-19^56^. Evidence-based life skills training (e.g., support for stress management, resilience training, sleep quality improvement) may need to be considered. Moreover, we observed that non-Hispanic Whites were more likely to experience poor mental health than non-Hispanic Black/African Americans, Asians, and Hispanics. This is in contrast to prior findings that found Black/African Americans to have poorer mental health^3,57,58^. Previously, stressful life events, including health, financial, or job problems in the past 30 days, had stronger associations with depression among non-Hispanic White men than non-Hispanic Black/African Americans in a national survey^59^. Thus, it is possible that non-Hispanic Whites were more vulnerable to poor mental health during the early COVID-19 pandemic, although further investigations in the extended and post-pandemic period are warranted to determine whether these differences persist.

Last, those who had chronic lung disease were more likely to have poor mental health, a finding that has been reported previously and is likely related to the poor quality of life among those with chronic lung disease^60,61^. Similarly, we also observed that those with poor general health status were more likely to have poor mental health, which is consistent with a previous report that considered depression and anxiety among cancer survivors^25^. Given that COVID-19 is a respiratory infectious disease, which disproportionately affected those with compromised health status, our finding highlights a vulnerable group to target for improving mental health.

Our study has some limitations. First, we were not able to confirm the direction of associations with factors in mental health as we used cross-sectional survey data. Second, even though HINTS data are high-quality and national, they might have some inevitable weaknesses originated from self-reporting, including reporting bias. For example, people might not report mental distress intentionally (hesitancy) or unintentionally (lack of awareness or knowledge). Third, we were not able to account for other cancer-related clinical information (e.g., cancer status, recurrence) as the HINTS did not collect this information. Fourth, the COVID-19 (2020) sample size was smaller than the pre-COVID-19 (2017–2019) sample size, as 2020 was the only available COVID-19 data from the HINTS. Lastly, COVID-19 data were collected from February to June 2020, hence the findings will need to be interpreted in the context of the early pandemic.

Despite these limitations, this study is among the first studies, to our knowledge, that examined the impact of the COVID-19 pandemic and cancer survivorship on mental health taking into consideration patient-provider communication at the population-level. The associations of PCC quality and digital device use for patient-provider communications with mental health have been rarely studied in spite of its potential role in online mental health care. Thus, our findings serve as a basis for future studies examining the dynamics of online health activities, the quality of communication, and mental health, as the use of online tools become widespread in health care, including telehealth.

A high proportion of adults in our study experienced poor mental health prior to and during the early COVID-19 in the U.S., yet neither the pandemic, nor cancer survivorship was related. Instead, OPPC use and lower socioeconomic status showed strong associations with poor mental health. Our findings highlight the importance of targeted approaches for these vulnerable subgroups, such as through partnering with communities or local governments, involving related stakeholders, or applying life skills training.

### Supplementary information

Reporting Summary

Supplemental Table 1

## Data Availability

The data used for this study is publicly available at https://hints.cancer.gov/data/download-data.aspx.

## Code availability

The code used to analyze the data is available from the corresponding author upon reasonable request.
