# Supplementary material for: Impact of COVID-19, cancer survivorship and patient-provider communication on mental health in the US Difference-In-Difference
Source: Npj Ment Health Res. 2023 Aug 30;2:14. doi: 10.1038/s44184-023-00034-x (PMC10955924; doi:10.1038/s44184-023-00034-x)
Supplement: Supplementary file 2 — Supplemental Table 1 [file 44184_2023_34_MOESM2_ESM.pdf]

Supplemental Table 1. The prevalence of poor mental health among cancer survivors and U.S. adults without a history of cancer in pre-COVID-19 (2017-19) and COVID-19 (2020).

	Cancer survivors		Adults without a history of cancer	
	Pre-COVID-19 N=1,953	COVID-19 N=626	Pre-COVID-19 N=10,124	COVID-19 N=3,168
	Weighted % (SE)	Weighted % (SE)	Weighted % (SE)	Weighted % (SE)
Average prevalence (%)[a]	37.4	41.9	38.4	40.2
**Age (years)**				
18-34	**71.5 (15.3)**	32.2 (20.7)	**45.1 (2.1)**	**47.0 (3.7)**
35-49	**50.4 (6.0)**	**65.5 (10.4)**	**39.2 (1.6)**	**44.4 (2.8)**
50-64	35.8 (3.0)	**43.9 (7.1)**	37.9 (1.4)	37.0 (2.7)
65-74	32.1 (2.8)	34.7 (4.9)	28.7 (1.4)	27.6 (2.5)
≥75	33.1 (2.9)	31.5 (4.9)	26.0 (1.8)	27.2 (4.0)
	p=.019	p=.043	p<.0001	p<.0001
**Gender**				
Female	**42.5 (2.4)**	**50.2 (4.4)**	**44.2 (1.0)**	**44.9 (2.2)**
Male	30.6 (3.1)	31.1 (5.6)	32.7 (1.5)	35.4 (2.3)
	p=.002	p=.004	p<.0001	p=.007
**Race/Ethnicity**				
Non-Hispanic White	35.5 (2.0)	43.9 (4.2)	40.1 (1.2)	40.0 (2.1)
Non-Hispanic Black/African American	**55.2 (9.0)**	28.3 (9.4)	35.3 (2.3)	39.6 (3.7)
Hispanic	**44.1 (7.6)**	37.8 (13.0)	36.2 (2.0)	**42.8 (2.9)**
Non-Hispanic Asian	9.6 (5.9)	33.2 (18.0)	33.0 (3.8)	22.2 (5.2)
Others	29.1 (9.5)	24.9 (19.5)	38.6 (4.6)	**60.6 (7.4)**
	p=.008	p=.086	p=.077	p=.006
**Education**				
Less Than High School	**69.4 (8.6)**	66.1 (13.1)	**48.2 (3.2)**	42.8 (5.5)
High School Graduate	**42.2 (3.8)**	43.9 (7.6)	36.9 (1.8)	43.3 (3.7)
Some College	34.6 (2.8)	41.4 (6.3)	**40.4 (1.6)**	40.9 (2.8)
College Grad or More	29.2 (2.3)	36.0 (5.5)	34.7 (1.1)	36.6 (2.0)
	p=.0002	p=.144	p=.0006	p=.232
**Household income**				
< $20,000	**59.2 (4.8)**	54.9 (7.6)	**54.1 (2.4)**	**59.4 (3.1)**
$20,000 to < $35,000	**45.4 (4.2)**	37.8 (9.7)	**41.5 (2.4)**	**44.2 (4.3)**
$35,000 to < $50,000	**41.4 (6.6)**	45.3 (9.9)	**44.5 (2.9)**	38.6 (4.9)

$50,000 to < $75,000	31.4 (4.0)	38.3 (6.8)	33.7 (2.2)	37.3 (4.0)
≥ $75,000	25.8 (2.2)	36.9 (5.6)	30.5 (1.2)	34.6 (2.1)
	p<.0001	p=.213	p<.0001	p<.0001
**Marital status**				
Married	31.4 (2.1)	37.9 (4.7)	32.2 (1.0)	34.1 (1.6)
Unmarried	**47.3 (3.2)**	49.2 (5.6)	**45.8 (1.6)**	**47.4 (2.7)**
	p<.0001	p=.092	p<.0001	p=.0003
**Health insurance**				
Private/employment	32.8 (3.3)	40.9 (6.0)	36.0 (1.1)	37.5 (2.0)
Medicare	30.5 (2.3)	29.7 (4.9)	28.7 (1.6)	32.5 (3.7)
Medicaid	**60.3 (6.1)**	**79.4 (7.0)**	**51.6 (2.0)**	**49.7 (3.0)**
Tricare, VA, IHS	**37.6 (5.6)**	37.5 (8.8)	37.1 (3.6)	**45.5 (5.2)**
Others	**40.7 (5.1)**	39.0 (8.6)	**44.4 (2.9)**	**50.1 (5.1)**
	p=.003	p=.005	p<.0001	p=.0007
**Rurality of residence**				
Metropolitan	37.2 (2.1)	39.9 (4.1)	37.8 (1.0)	40.5 (1.5)
Micropolitan	31.6 (4.7)	39.8 (12.0)	45.7 (3.3)	34.9 (6.0)
Small town	49.4 (8.3)	69.4 (14.8)	34.2 (3.7)	46.1 (8.6)
Rural	44.9 (9.6)	50.9 (13.0)	40.7 (3.9)	38.7 (11.7)
	p=.254	p=.375	p=.112	p=.761
**General health status**				
Excellent/great	29.0 (1.9)	37.5 (4.6)	33.9 (0.9)	36.8 (1.7)
Fair/poor	**62.3 (3.6)**	**56.4 (6.4)**	**65.1 (2.0)**	**63.2 (4.0)**
	p<.0001	p=.016	p<.0001	p<.0001
**Chronic medical condition**				
Diabetes	**44.0 (3.8)**	36.4 (5.9)	**47.0 (1.9)**	**46.8 (3.4)**
No diabetes	35.3 (2.1)	43.5 (4.1)	36.8 (1.0)	38.8 (1.6)
	p=.038	p=.328	p<.0001	p=.044
High blood pressure	**41.8 (2.4)**	44.7 (4.9)	**41.3 (1.3)**	**44.6 (2.5)**
No high blood pressure	32.2 (3.1)	38.6 (5.7)	36.9 (1.1)	38.0 (1.8)
	p=.015	p=.388	p=.010	p=.035
Heart disease	40.4 (5.0)	**58.4 (8.0)**	**44.5 (2.9)**	41.0 (4.3)
No heart disease	36.8 (2.0)	39.5 (4.1)	38.0 (0.9)	40.1 (1.6)
	p=.481	p=.043	p=.044	p=.871
Lung Disease	**54.8 (4.1)**	**55.8 (7.5)**	**56.6 (2.2)**	**53.9 (3.6)**
No lung disease	33.9 (2.1)	38.9 (4.5)	36.2 (0.9)	38.3 (1.7)
	p<.0001	p=.048	p<.0001	p=.0007
**Time since diagnosis**				

Less than 1 year	43.4 (7.2)	44.2 (7.2)	–	–
2-5 Years	37.3 (3.4)	30.8 (7.3)	–	–
6-10 Years	35.0 (4.1)	44.7 (7.7)	–	–
11 Years or more	36.5 (2.7)	43.9 (5.9)	–	–
	p=.742	p=.461		
**Online PPC**				
Email/Internet communication	37.7 (3.2)	40.4 (5.2)	**42.3 (1.4)**	**45.9 (2.2)**
No Email/Internet communication	37.1 (2.3)	43.6 (4.6)	36.0 (1.1)	35.1 (2.0)
	p=.879	p=.585	p=.0009	p=.001
Tablet/Smartphone for discussion	**44.4 (3.9)**	46.5 (6.6)	**45.2 (1.5)**	**46.6 (2.7)**
No Tablet/Smartphone for discussion	**34.3 (2.1)**	39.0 (4.3)	35.4 (1.1)	36.7 (1.8)
	p=.030	p=.293	p<.0001	p=.006
EHR message	39.8 (3.1)	34.3 (5.9)	40.5 (2.0)	**46.9 (2.7)**
No EHR message	36.8 (2.1)	45.4 (4.1)	37.9 (1.0)	38.2 (1.5)
	p=.419	p=.051	p=.259	p=.006
	Cancer survivors		Adults without a history of cancer	
	Pre-COVID-19	COVID-19	Pre-COVID-19	COVID-19
**PCC**[b]	**Mean (SE)**	**Mean (SE)**	**Mean (SE)**	**Mean (SE)**
Composite score	81.0 (0.9)	79.3 (1.3)	79.9 (0.4)	79.8 (0.8)

a. Poor mental health outcome was determined if either having depression/anxiety or psychological distress. The prevalence is presented by each group; b. PCC score ranges from 0 (sub-optimal) to 100 (optimal), higher is better; **Bold** means higher than the average prevalence in each group. p-value was obtained from a chi-square test; Abbreviations (VA: Veterans Affairs; IHS: Indian Health Services)
